# Robot Comedy Lab: experimenting with the social dynamics of live performance

**DOI:** 10.3389/fpsyg.2015.01253

**Published:** 2015-08-25

**Authors:** Kleomenis Katevas, Patrick G. T. Healey, Matthew Tobias Harris

**Affiliations:** Cognitive Science Research Group, School of Electronic Engineering and Computer Science, Queen Mary University of LondonLondon, UK

**Keywords:** human robot interaction, affective computing, humanoid robots, live performance, social signals

## Abstract

The success of live comedy depends on a performer's ability to “work” an audience. Ethnographic studies suggest that this involves the co-ordinated use of subtle social signals such as body orientation, gesture, gaze by both performers and audience members. Robots provide a unique opportunity to test the effects of these signals experimentally. Using a life-size humanoid robot, programmed to perform a stand-up comedy routine, we manipulated the robot's patterns of gesture and gaze and examined their effects on the real-time responses of a live audience. The strength and type of responses were captured using SHORE™computer vision analytics. The results highlight the complex, reciprocal social dynamics of performer and audience behavior. People respond more positively when the robot looks at them, negatively when it looks away and performative gestures also contribute to different patterns of audience response. This demonstrates how the responses of individual audience members depend on the specific interaction they're having with the performer. This work provides insights into how to design more effective, more socially engaging forms of robot interaction that can be used in a variety of service contexts.

## 1. Introduction

Not everyone knows how to tell a joke. A good joke depends as much on the quality of the delivery as it does on the quality of the material. Intonation, posture, gaze, gesture, expression, and timing all contribute to successful comic delivery. Moreover, effective delivery is not just a matter of what the speaker does, it also depends on the reciprocal dynamics of the speaker–listener interaction. The fluency of speakers' performance in conversation depends on the moment-to-moment responsiveness of their audience and, in turn, on the speakers' ability to concurrently accommodate and adjust to these responses while they are speaking (Goodwin, 1979; Bavelas et al., 2000). If addressees appear to be bored or distracted, speakers become disfluent. Conversely, an appropriately timed smile or raised eyebrow by an addressee provides useful feedback that speakers can use to adapt their message.

Our basic hypothesis is that these interactional dynamics should be just as important to the mass interaction involved in performing in front of a live comedy audience as they are to telling a joke to a friend. Ethnographic studies of stand-up comedy and street performance support this idea. Gardair (2013) demonstrated the pervasive relevance of interaction to the achievement of a successful street performance. Street performances are actively established and managed using patterns of interaction similar to those used to establish and maintain conversational clusters or “F-formations” (Kendon, 1990). Street performers use variations of body position, orientation and gaze to manage engagement and define the performance space (Gardair et al., 2011). They invest considerable effort in orchestrating, explicitly eliciting and in some cases actively training the audiences' responses. This process appears to be key to the development of a collective sense of audience membership and often takes up more than 90% of the performance time. It also appears to play a critical role in obtaining money from the audience (Gardair, 2013).

Rutter (1997, 2000) argued that stand-up comedy is also defined by interaction: the performance is an interactive organization and delivery of material constantly informed by audience responses; for an audience, becoming involved in the developing flow of the act engenders not just an active and responsive manner but one where all can be held to account. We assume that it is these interactional processes that contribute to the distinction that performers make between “good” and “bad” audiences for the same performance. Furthermore, it is the same processes that underpin people's experience of moments of “crackle”, “movement” and “lift”, or “drop” and “drift” that are part of the practical language of performance (Healey et al., 2009).

Embodied robots provide a unique opportunity to experiment with these interactional processes by enabling the introduction of controlled manipulations directly into a live performance. Although robots have the disadvantage of eliciting responses that may be different from those of a human performer, they can hold the “content” of the routine constant (e.g., the prosody, semantics and syntax of the jokes) while selectively manipulating aspects of delivery (e.g., body orientation, gaze, and gesture). This strategy of using embodied robots as tools for human interaction experiments has precedents in work by MacDorman and Ishiguro (2006), Sidner et al. (2005), and Knight and Simmons (2013). These studies use robots to experiment with different aspects of overt robot behavior, including gaze and gesture, as a means of probing the detailed organization of social interaction. This enables direct comparisons of the effects of different behaviors on interaction and can provide a principled basis on which to design robots that can engage successfully with humans.

Previous work has also specifically made use of embodied robots to tell jokes. Hayashi et al. (2005) created a robot–robot dialogue system so that two robots could enact Japanese “Manzai” routines in front of an audience. Although the robot's movements were scripted, the timing of their jokes was sensitive to audience responses. Sjöbergh and Araki (2009) used the Robovie-i platform to show that the same jokes delivered by a robot are rated as funnier on average than when delivered in text form only. They also showed a larger effect of robot responses (positive or negative) on perceived funniness of jokes. Knight et al. (2011) used the Nao robot to explore how the choice of jokes from a larger repertoire could be customized according to the strength of audience responses. These studies effectively held the non-verbal delivery of each joke constant.

Here we use a robot to explore specific non-verbal elements of performer-audience interaction in comic delivery. In order to motivate the choice of experimental manipulations we briefly describe a pilot study of a stand-up comedy performance. Building on the observations from this study and previous ethnographic work on performer-audience interaction, we describe the “Comedy Parser” system we developed to support performative gaze and gestures in a commercial robot platform (Katevas et al., 2014). The impact of these manipulations was analyzed in a live performance experiment conducted over two nights at the Barbican Centre in London. As far as we are aware this is the first attempt to use a robot to probe the moment-by-moment, embodied aspects of how stand-up comedians “work” an audience.

## 2. Comic observations

A pilot observational study was made using video data taken from a “Comedy Lab” hosted in the Performance Lab at Queen Mary University of London. The aim of this study was to extend previous ethnographic observations of performer-audience interaction in street performance (Gardair et al., 2011; Gardair, 2013) to the specific context of stand-up comedy. In particular, to get a more detailed sense of some of the elements of non-verbal delivery required from a robot to “read” and respond to an audience.

**Figure 1:**
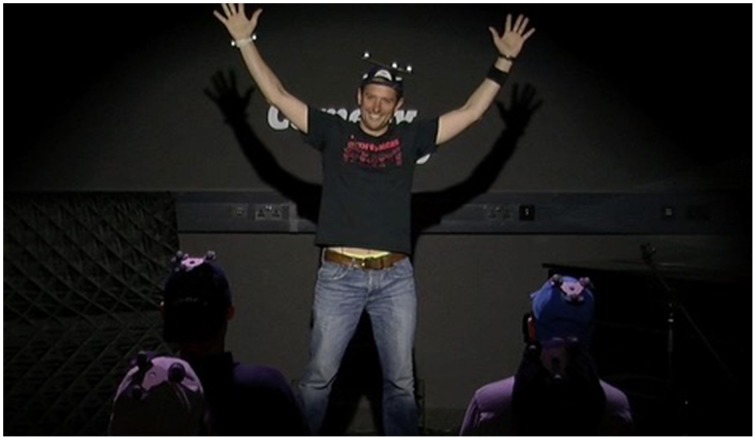
**Comedy Lab with Stuart Goldsmith, at Queen Mary University of London**.

The Comedy Lab session featured live stand-up performances by two professional comedians: Tiernan Douieb (compère) and Stuart Goldsmith (main act), with 25 audience participants recruited through social media channels. Douieb provided a 5 min “warm-up” and then introduced Goldsmith who did a 15 min set (Figure 1). Full-HD audio-visual recordings were made using fixed cameras approximating the audience's view of the performer and the performer's view of the audience. All data collection and analysis was made with informed consent and approved by the Queen Mary University of London research ethics committee (Reference: QMREC1199b).

The video recordings of the performer and audience were imported into ELAN, a multimedia annotation tool (Wittenburg et al., 2006). A simple qualitative analysis of the performer's and audiences' use of non-verbal signals was made using multiple passes over the tape using ELAN to control speed of playback and to code significant events.

### 2.1. Comic delivery

Patterns of performer-audience interaction are complex and a detailed analysis is outside the scope of the current paper, however several observations are relevant to the robot behaviors described below. First, the gaze of the performer was predominately either to the floor or to an audience individual. Second, the performer's gaze tended to shift at the end of every sentence, and sometimes between phrases, in a pattern similar to that observed in conversation where speakers use gaze to elicit responses from their addressees (Kendon, 1967; Goodwin, 1979). The performer would also focus their gaze on an audience participant accompanied by a pointing gesture and reference to the participant in the talk, making it clear that a specific audience member was being addressed.

Punchlines were typically distinguished by faster delivery and a short pause and change of gaze. In such cases, the change of gaze was always onto an audience member. In addition, the performer usually followed their punchlines with a smile and sometimes laughter. Resumption following a punchline appeared to be primarily contingent on the audience response. The duration of pause after the punchline was determined by whether and how quickly laughter ensued. If the laughter was significant, the performer would remain silent until the laughter started to subside—a pattern also noted for Japanese Manzei performances by Hayashi et al. (2005). Audience laughter was marked not only by facial displays but also large visible body movements of the head and upper body. Audience members directed their gaze mostly at the performer, occasionally to each other or the floor.

As with street performance, the comedians also occasionally used large gestures (Figure 1) designed to promote a stronger or more prolonged audience response similar to the applause elicitation gestures described by Gardair (2013). Another interesting shared feature with street performance is the stand-up comics' use of explicit commentaries on the character of audience responses as a way to generate more active engagement, e.g., complaining about isolated or weak laughter (Gardair et al., 2011; Gardair, 2013).

Drawing on these findings our experiment manipulated the robot's gaze at audience members and the production of specific performative gestures (see Section 3.3.2) and then assesses their impact on the audience responses.

## 3. Experimental study

### 3.1. Study overview and predictions

**Figure 2:**
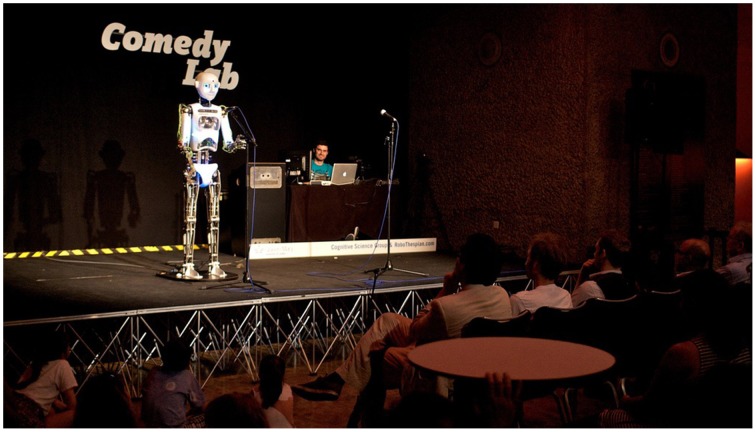
**Comedy Lab with RoboThespian™ at the Barbican Centre in London**.

“Comedy Lab: Human vs. Robot” was conceived as a performance experiment, carried out in an arts venue in front of a live audience (Figure 2). The basic rationale was to use a robotic performer to perform a predetermined comedy script while different aspects of the delivery were manipulated and live audience responses gathered for analysis.

Our first manipulation involves gaze: we dynamically allocated different audience members as gaze targets for the robot during the performance. Although it seems intuitive that people simply smile when they are happy, displays of positive affect are conditioned by social context. Following Bavelas et al. (1986) we assumed that even in the somewhat anonymized context of a live performance, the overt responses and facial expressions produced by audience members are communicative displays designed for specific recipients. This is a relatively strong assumption because we are proposing that the principal recipient of the audiences' facial displays in this context is the robot. This lead to the prediction that audience members should display more positive affect when they believe the robot is attending to them and less when they believe it is not. Note that this is independent of how funny they find the jokes themselves.

Our second manipulation involves gesture: a number of special gestures were programmed as exceptions to the default “canned” movements delivered by the robot platform. Drawing on our pilot observational study (Section 2.1) and observational studies of street performances (Gardair, 2013), we opted to test the effects of four specific gestures that appeared to be designed to elicit positive audience responses (illustrated in Section 3.3.2). The first of these was a raised arm “welcome” gesture, the second an “emphasis” gesture, the third a pointing gesture, and the fourth an applause eliciting gesture. If these gestures are effective in promoting stronger engagement this should be evident in their effects on measures of positive affect in the audience responses.

We also tested our basic assumption that the experiment succeeds in creating a credible stand-up performance by assessing whether the jokes themselves, written by the compère Douieb, elicit positive responses. Although not central to the questions about delivery that are the main concern of this paper, this is an important issue for the validity of the study. It also provides a test of whether people responded to the specifics of the performance rather than adopting a generic positive (or sceptical) attitude due to the novelty of seeing a robot performing. Other studies showed robot performers may elicit similar or stronger positive responses than their human counterparts (Hayashi et al., 2005).

### 3.2. Materials and methods

Before proceeding to describe the design of the study we first introduce the computer vision software and robot platform that were used in this study.

#### 3.2.1. Measures of audience response

To obtain fine-grained real-time response measures and automatic measures of facial display and position, we used sentiment analysis techniques developed in computer vision research. Fraunhofer SHORE™ (Sophisticated High-speed Object Recognition Engine) was selected for this purpose.

Provided with video imagery, SHORE™ detects faces within each frame and provides properties for each of them. In our tests we found SHORE™ able to detect audience faces when seated under low (but under our control) lighting conditions and filmed from the front, and able to do so in real-time. The properties the software produces for each identified face, and so makes available for experimental measures, are the following:

- Location of the face in the space.
- Position of the eyes, nose and mouth.
- Gender classification (“Male”, “Female”, or “Unknown”).
- Age estimation in years.
- Facial expression recognition, expressed as percentages of “Happy”, “Sad”, “Angry”, and “Surprised”.
- Identify whether the eyes are open or closed.
- Identify how much the mouth is open.
- Detection of up to 60° of face rotation.

Most of the above features have been validated using external data sets (Ernst et al., 2009). The face detection has been validated using the CMU+MIT data sets and showed good accuracy relative to other classification methods (91.5% detection rate with a 1 in 10 miss rate). The gender classification has been validated using the BioID data set (94.3% recognition rate) as well as the Feret fafb data set (92.4% recognition rate). Finally, the happiness analyzer has been validated on the JAFFE data base (95.3% recognition rate). Note that none of these test datasets were used as training sets for the framework. Further information can be found on the Fraunhofer IIS website: http://iis.fraunhofer.de.

#### 3.2.2. The robot platform

RoboThespian™ is a humanoid robot designed for interaction in public places created by Engineered Arts Ltd. (see Figures 2, **4**). Following a human body model, it consists of a robotic head, two arms with hands, the robot's torso as well as the two legs. The robotic head has two rectangular LCD screens for eyes as well as embedded LED lighting in the cheeks that allows it to make facial expressions as it talks. The mouth can only move vertically, a process that is automated and synchronized with the speech engine. The two arms and hands can move fast and fluently, while the torso's movement is relatively limited and slow. The robot cannot walk by itself as it only has passive leg movement. It also uses the Acapela Text-To-Speech engine from Acapela Group Babel Technologies SA, providing voice synthesis in customizable voices, as well as control over speed, timing, volume, and shape (sound pitch).

To provide the robot platform with the capabilities required for the experiment we built a “Comedy Parser” system that controls the robotic behavior including its interaction with the audience (Katevas et al., 2014). Provided with a specially marked-up script, it delivers the content while enacting the behaviors described in Section 2. It uses SHORE™ computer vision software to analyze the audience in real-time and identify each person's location in the space as well as to capture characteristics such as gender, age and moment-by-moment display of “happiness”. The complete source-code, licensed under an MIT License, is available at https://github.com/minoskt/ComedyParser.

#### 3.2.3. Procedure

Two performances were staged as part of the “Hack the Barbican” event at 6 p.m. on 7th and 8th of August 2013 at the Barbican Centre in London. The club stage that was used is freely accessible to the public. Each performance comprised the compère's warm up, and the (human) comedian's act followed by the robot act. The compère's warm-up lasted approximately 10 min, the comedian's lasted 13 min, and the robot's 8 min. Two professional stand-up comedians, Tiernan Douieb and Andrew O'Neill, were recruited for the compère and comedian roles, respectively. This format was used both to widen the appeal of the event and to help create a more convincing stand-up comedy context. Although the compère and comedian made normal stage entries and exits the robot cannot walk and therefore its position and the control desk were fixed throughout (see Figure 2). During the robot's performance, an experimenter monitored the control equipment, visible to the side at the rear of the stage.

**Figure 3:**
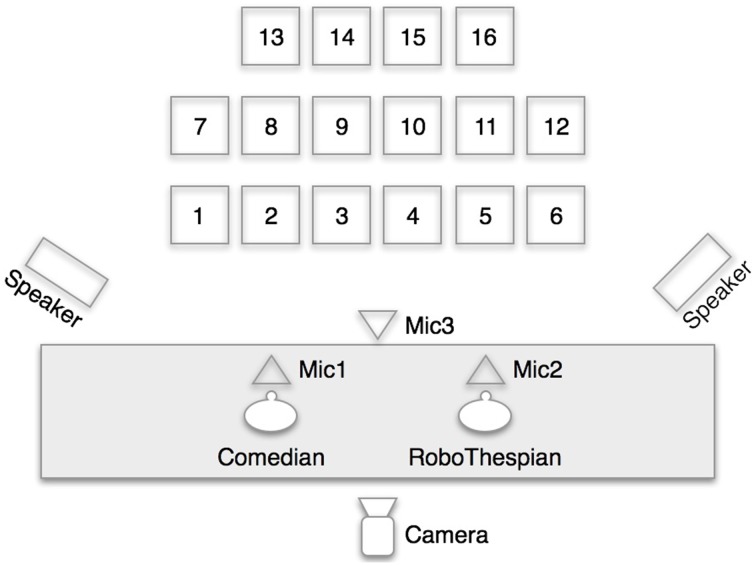
**Barbican Comedy Lab configuration**.

Figure 3 shows the configuration of the staging, with the seat placement, the position of the performers, as well as the position of the two speakers, the tracking camera and the three directional microphones. The tracking camera was an inconspicuous Gig-E Vision camera positioned high at the back of the stage with a field of view that encompassed the seated area.

An audio-visual recording of each performance was obtained by placing an HD video camera toward the back of the audience area. SHORE™ software analyzed video imagery from the tracking camera and passed the output to the Comedy Parser. Comedy Parser also archived all dynamic aspects of the performance, in particular the robot's gaze and point.

Participants were informed that they were being captured on video for research purposes and all data capturing and handling procedures were audited by the Queen Mary University of London research ethics committee (Reference: QMREC1199b).

#### 3.2.4. Participants

Audience participants were recruited by advertising “Comedy Lab” through social media channels of the two performers, the venue (The Barbican Centre, London), research group (Cognitive Science, Queen Mary University of London) and “Hack the Barbican”. The following context was provided in the advert:

What makes a good performance? By pitting stand-up comics Tiernan Douieb and Andrew O'Neill against a life size robot in a battle for laughs, researchers at Queen Mary University of London hope to find out more— and are inviting you along.

A collaboration between the labs of Queen Mary's Cognitive Science Research Group, RoboThespian's creators Engineered Arts, and the open-access spaces of Hack The Barbican, the researchers are staging a stand-up gig where the headline act is a robot as a live experiment into performer-audience interaction.

This research is part of work on audience interaction being pioneered by the Cognitive Science Group. It is looking at the ways in which performers and audiences interact with each other and how this affects the experience of “liveness”. The experiment with RoboThespian™ is testing ideas about how comedians deliver their material to maximize comic effect.

Approximately 50 people attended each performance on each night. Data from SHORE™ were captured for 22 people for the first night (15 men and 7 women between the ages of 28 and 64 years, *M* = 46.4, *SD* = 8.0) and 19 for the second night (13 men and 6 women between the ages of 27 and 60 years, *M* = 46.2, *SD* = 8.1).

### 3.3. Results

The measures of “Happiness”, “Anger”, “Surprise”, and “Sadness” produced by SHORE™ showed substantial inter-correlations. For example, in our data “Happiness” and “Sadness” are negatively correlated for: Pearson's *r*_(121)_ = −0.484, *p* < 0.01 (Note: *N* = 121 because there are three measures for each of 48 people corresponding to Before, During and After a punchline, discussed in more detail below); and so are “Happiness” and “Anger”: Pearson's *r*_(121)_ = −0.433, *p* < 0.01. These correlations make these measures partially redundant and we therefore report results only for the “Happiness” measure in the following analysis.

Throughout we report computed probabilities for completeness but adopt a criterion level of *p* < 0.05 for inferences. We use Generalized Linear Mixed Model (GLMM) analyses to model the combined random effects, categorical and interval fixed effects and repeated measures involved in the audience responses measured in this study.

#### 3.3.1. Punchlines

To test if audience members respond selectively to the jokes, their facial displays of “Happiness” were averaged over three “Response Phases”: “Before”, “During”, and “After” defined as, 2 s before the punchline, the duration of the punchline delivery and 2 s after.

Average “Happiness” displayed by the audience was analyzed in a GLMM using a Linear Model. This treated Response Phase (Before/During/After) as a fixed factor and Audience Member nested within Night as random factors. It shows a main effect of Response Phase [*F*_(2, 120)_ = 5.66, *p* < 0.01]. Planned, pairwise comparisons show that people displayed more happiness after the punchlines than before them [*t*_(120)_ = 3.32, *p* < 0.01] or during them [*t*_(120)_ = 2.67, *p* = 0.01] but no difference in displayed happiness before and during the punchlines [*t*_(120)_ = −0.86, *p* = 0.39]. The estimated means and standard errors are summarized in Table 1. Fixed (*B*) coefficients provide estimates of effect size: After = 2.3, (95% CI lower = 0.6, upper = 4.0); Before = −0.59, (95% CI lower = −1.9, upper = 0.7). *During* is the reference category.

**Table 1:** **Estimated means and standard errors for “Happiness” before, during and after punchlines**.

**Response phase**	**Estimated mean**	**Std. error**
Before	44.2	3.17
During	44.8	3.13
After	47.1	3.12

#### 3.3.2. Gestures

**Figure 4:**
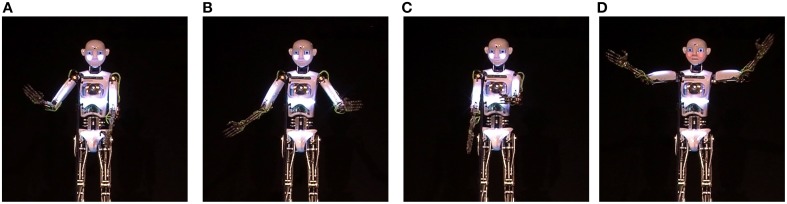
**Performative Gestures used during the live performance**. **(A)** “Welcome” gesture, **(B)** Reprise “I said hello” gesture, **(C)** Pointing gesture, **(D)** Applause elicitation gesture.

During each performance, RoboThespian™ used four specific performative gestures. Due to timing issues, the first “welcome” gesture (Figure 4A) was not obvious to the participants as they were still applauding, welcoming RoboThespian™ on stage. Consequently this gesture is excluded from the analysis. The following three gestures are analyzed:

1. *Gesture B:* A reprise “I said hello” gesture that emphasizes the expected return of greetings suggested by Gardair's (2013) analysis of street performances (Figure 4B).
2. *Gesture C:* A pointing gesture while saying “you go first”, inspired by our observational study of stand-up comedy (Figure 4C).
3. *Gesture D:* The applause elicitation gesture “Thank you, and good night,” inspired by Gardair's (2013) analysis of street performances (Figure 4D).

As the gestures are qualitatively different in their effects we analyze them separately.

##### 3.3.2.1. Gesture B

A GLMM Linear Model analysis of average displayed Happiness in response to Gesture B with Response Phase (Before vs. During vs. After) as a fixed factor and Night (1 vs. 2) and Audience Member as random factors showed no main effect of Response Phase [*F*_(2, 99)_ = 1.63, *p* = 0.20]. Planned pairwise comparisons also showed no difference between the different response phases {Estimated Means: Before = 37.0, During = 42.6, After = 41.4; Pairwise Comparisons: Before vs. During: *t*_(99)_ = −1.71, *p* = 0.09, Before vs. After = [*t*_(99)_ = −1.34, *p* = 0.18], During vs. After [*t*_(99)_ = 0.34, *p* = 0.73]}.

##### 3.3.2.2. Gesture C

A GLMM analysis with the same factors as above showed a different pattern of responses. For Gesture C there was a significant main effect of Response Phase [*F*_(2, 106)_ = 6.11, *p* < 0.01]. The estimated means are provided in Table 2. Pairwise comparisons show that displayed happiness increased during and immediately after the production of Gesture C but were not reliably different while the gesture was produced and immediately after: Before vs. During: *t*_(106)_ = −3.23, *p* < 0.01, Before vs. After = [*t*_(106)_ = −3.1, *p* < 0.01], During vs. After [*t*_(106)_ = 0.44, *p* = 0.66]. Fixed (*B*) coefficients: Before = −8.7, (95% CI lower = −14.4, upper = −3.14); During = 0.44, (95% CI lower = −3.8, upper = 6.0). After is redundant.

**Table 2:** **Estimated means and standard errors for “Happiness” before, during and after execution of Gesture C**.

**Response phase**	**Estimated mean**	**Std. error**
Before	42.3	4.3
During	52.2	4.1
After	51.1	4.0

##### 3.3.2.3. Gesture D

The parallel GLMM analysis for Gesture D shows no main effect of Response Phase: *F*_(2, 92)_ = 2.13, *p* = 0.13. Planned pairwise comparisons showed no reliable differences between the three response phases: Before vs. During *t*_(92)_ = −1.11, *p* = 0.27; Before vs. After *t*_(92)_ = 0.44, *p* = 0.66 During vs. Before *t*_(92)_ = 1.12, *p* = 0.27.

Overall, only Gesture C produced a reliable positive response. The three different Gestures are, of course, designed to achieve different effects. Gesture B is designed primarily to prompt applause and cheering. Gesture C works to underline the point of a joke and responses seem to be closely tied to the timing of the gesture delivery. For Gesture D the aim is to elicit applause. Unfortunately we do not have robust quantitative measures of these different responses.

#### 3.3.3. Gaze

A total of 14 unique individuals in the audience were randomly fixated a total of 153 times by the robot over the two nights. Three people were fixated only once and are excluded from the analysis.

The effect of Gaze on displayed “Happiness” is analyzed in a GLMM linear model with Audience Member as a random factor and Gaze Phase (2 s Before, During and 2 s After) a fixed factors. The robot's fixation points were not exact so the distance in pixels between a participant's actual location in the video and the fixation point of the robot is included as a co-variate. This analysis shows a main effect of Gaze Phase [*F*_(2, 238)_ = 14.5, *p* < 0.01] and a main effect of Distance [*F*_(1, 242)_ = 5.19, *p* < 0.05]. The estimated means are provided in Table 3. Planned pairwise comparisons of Gaze Phase show no difference in the fixated person's displayed “Happiness” before and during the fixation [Before vs. During *t*_(238)_ = 0.02, *p* = 0.99] but a significant drop afterwards [After vs. Before *t*_(238)_ = −4.68, *p* < 0.01, After vs. During *t*_(238)_ = −4.96, *p* < 0.01]. Fixed (*B*) coefficients for Gaze Phase: Before = 5.8, During = 5.8, After = 3.2.

**Table 3:** **Estimated means and standard errors for “Happiness” before, during and after robot gaze**.

**Response phase**	**Estimated mean**	**Std. error**
Before	45.1	4.3
During	45.1	4.3
After	42.1	4.3

The fixed coefficient (*B*) for the distance co-variate of −0.12 additionally showed that the further an audience member's face was from the center of the robot's fixation point the lower the estimated facial display of “Happiness”.

#### 3.3.4. Human vs. Robot

Although direct comparison of the human and robot performers was not part of the original study design (and is in some respects problematic see Section 4 below) it is interesting to compare the overall “Happiness” response evoked by the compère, comedian and robot.

A *post-hoc* GLMM linear model analysis of average percentage happiness of each audience member on each night with Performer (Compère vs. Human Comedian vs. Robot) as a fixed factor and Audience Member and Night as a random factors shows a main effect of Performer [*F*_(2, 227)_ = 9.37, *p* < 0.01]. Planned pairwise comparisons show people responded more positively to the human comedian than the compére [*t*_(227)_ = 4.33, *p* < 0.01] but no other comparisons were significant [Compère vs. Robot *t*_(227)_ = 1.85, *p* < 0.1; Comedian vs. Robot *t*_(227)_ = 1.37, *p* < 0.2]. As Table 4 shows, people's responses to the Robot were essentially intermediate between the two human performers.

**Table 4:** **Estimated means and standard errors for “Happiness” response to each performer**.

**Response phase**	**Estimated mean**	**Std. error**
Compére	38.2	4.1
Comedian	45.6	4.0
Robot	42.6	4.1

## 4. Discussion

At the broadest level these results demonstrate the viability of using embodied robots to study the social dynamics of live performance. The ability to make controlled, fine-grained manipulations of gaze and gesture while holding other aspects of performance constant creates exciting possibilities for future research that go beyond what is possible using human confederates; people are simply unable to selectively control their own performances to the same degree as a robot (Kuhlen and Brennan, 2013). Balanced against this are the issues that arise from the fact that the performer is plainly a robot.

Anecdotally, our observation and personal discussions with people afterwards suggested that audience members on the two nights of Comedy Lab found the jokes generally amusing despite the restricted prosody and cadence of the robot platform's speech synthesis. However, audience responses might have been biased by the novelty of the situation. For example, Hayashi et al. (2005) provided evidence that people are more sympathetic to a robot comedian than a human comedian, although in this work a live robot performance was compared with a recorded human performance. Audience bias might also run in the opposite direction; our audience was explicitly recruited for a robot vs. human “Comedy Lab” and contained some journalists and people with a technical interest in robotics. Consequently, they might be atypical of a stand-up comedy audience and more interested in the technical than the comic material. We note that both comedians said informally that they found the audience harder to engage than a typical stand-up comedy club.

The present study does not provide data that enables us to assess audience bias directly. The finding that people responded as positively to the robot as they did to the human stand-up suggests that any potential bias was limited. However, we note that this comparison is confounded by differences in, amongst other things, staging, materials and delivery. We avoid this problem here by focusing our analysis on the comparison of audience responses to the robot before, during and after the specific manipulated behaviors. This allows us to broadly discount the potential influence of people's generic dispositions toward robot performers; positive or negative.

Importantly, the results showed that audience responses are closely co-ordinated with the delivery of the punchlines and robot gaze. Specifically, displays of positive affect peaked just after the punchlines but declined after gaze. This showed that people were selectively responsive to both the content and delivery. Audience members appeared to be particularly sensitive both to whether the robot was looking at them and to the specific angle of the robot's gaze. The more closely they were fixated by the robot, the more positive affect they displayed. The robot was treated as a social agent that successfully elicited social response patterns typical of human interactions. This finding supports our hypothesis that performers use gaze as a means of eliciting audience responses (Kendon, 1967). It is also consistent with prior work that has noted the importance of social gaze in storytelling performances by embodied robots (Mutlu et al., 2006) and humans (Goodwin, 1979).

The pattern of results for the manipulated gestures is less clear. Only the pointing gesture (see Figure 4C) had a statistically significant effect. There may be several reasons for this. It might be due to a lack of measures appropriate to each gesture, e.g., the emphasis gesture may have caused a louder or more emphatic response that would not necessarily show up in the measures of facial affect. It might be due to problems in the execution of the gestures that made them difficult to interpret or it might be that the gestures simply do not function in the way we expected.

Overall, the results demonstrate a fine-grained link between specific aspects of delivery and specific audience responses. This is consistent with the general hypothesis that part of what underpins the experience of live performance is the social dynamics of audience-performer interactions (Rutter, 2000; Gardair et al., 2011; Gardair, 2013). As noted in the introduction, laughter, smiles and other displays of affect are themselves performances designed for audiences including our conversational partners (Kraut and Johnston, 1979; Bavelas et al., 1986; Fernández-Dols and Ruiz-Belda, 1995). The data presented here show how this use of displays of affect extends to live comedy audiences and, in particular, to specific moments of engagement between performers and individual audience members. Like the observational studies described in the introduction it provides evidence that performers modulate audience responses not only through large performative gestures but also through the use of fine-grained mechanisms such as eye contact. Moreover, it shows that these mechanisms lead to different patterns of response for different audience members. Understanding specific moment-by-moment processes that underpin these interactional dynamics is key to developing more compelling live experiences and more engaging robots.

This exploratory study needs to be replicated and extended. A larger repertoire of gestures and other non-verbal signals needs to be tested together with a richer set of response measures. An obvious limitation here is the possible influence of the specific audience and context. Testing alternative patterns of delivery across a wider range of audiences would help to establish how general these patterns are. Greater realism could be achieved by using motion capture sequences from a human comedian to drive the robot. These sequences could provide the basis for more naturalistic manipulations of different non-verbal elements of performance and would also support more credible robot-human comparison.

The Comedy Parser platform (Katevas et al., 2014) demonstrates how robot performances can use the computational vision capabilities provided by systems like SHORE™ to make the details of delivery contingent on how each individual in an audience is responding in real-time. We note that this goes beyond what a human comic can do. This approach can be extended to other modalities such as automatic, real-time audio processing to sense oral responses, applause and more subtle cues such as collective inbreaths or rustling paper. There is also potential for experimenting with speech rhythm and intonation. Alternative speech engines provide some interesting capabilities. For example, CereVoice is capable of changing of the voice's “mood” into “happy”, “calm”, or “joke” (Aylett and Pidcock, 2007).

## 5. Conclusion

This paper demonstrates how humanoid robots can be used to probe the complex social signals that contribute to the experience of live performance. Using qualitative, ethnographic work as a starting point we can generate specific hypotheses about the use of social signals in performance and use a robot to operationalize and test them. This can provide a principled basis on which to give humanoid robots the capabilities needed to interpret and respond to the social dynamics of massed audiences.

Moreover, this paper provides insight into the nature of live performance. We showed that audiences have to be treated as heterogeneous, with individual responses differentiated in part by the interaction they are having with the performer. Equally, performances should be further understood in terms of these interactions. Successful performance manages the dynamics of these interactions to the performer's- and audiences'-benefit.

## Author contributions

This work is based on research carried out for an advanced placement project by KK under the supervision of PH. Concept: PH, KK, MH. Observational study: MH, KK. Experimental design: PH. Engineering of robot platform: KK. Data collection and processing: KK, MH. Statistical analysis and interpretation: PH. Manuscript: PH, KK, MH.

### Conflict of interest statement

The authors declare that the research was conducted in the absence of any commercial or financial relationships that could be construed as a potential conflict of interest.
